# Social Psychology of Coronavirus Disease 2019: Do Fatalism and Comparative Optimism Affect Attitudes and Adherence to Sanitary Protocols?

**DOI:** 10.3389/fpsyg.2021.623005

**Published:** 2021-05-13

**Authors:** Trond Nordfjaern, Milad Mehdizadeh, Mohsen Fallah Zavareh

**Affiliations:** ^1^Department of Psychology, Norwegian University of Science and Technology, Trondheim, Norway; ^2^School of Civil Engineering, Iran University of Science and Technology, Tehran, Iran; ^3^Department of Civil Engineering, Faculty of Engineering, Kharazmi University, Tehran, Iran

**Keywords:** SARS-CoV-2, psychological factors, developing country, iran, fatalistic beliefs

## Abstract

The potential of mitigating the spreading rate and consequences of the coronavirus disease 2019 (COVID-19) currently depends on adherence to sanitary protocols (e.g., hand hygiene and social distancing). The current study aimed to investigate the role of fatalism and comparative optimism for adherence to COVID-19 protocols. We also tested whether these factors are directly associated with adherence or associated through attitudinal mediation. The results were based on a web survey conducted among university students (*n* = 370) in Tehran, Iran. The respondents completed a multidimensional measure of fatalism (general fatalism, internality, and luck) and measures of comparative optimism, attitudes toward COVID-19 health measures, and adherence. The estimated structural equation model explained approximately 40% of the total variance in attitudes toward COVID-19 protocols and adherence. As expected, high internality was associated with stronger adherence, whereas luck was associated with weaker adherence. Comparative optimism was more strongly associated with adherence than fatalism, and somewhat unexpectedly comparative optimism was associated with stronger adherence. Analyses of direct and indirect effects suggested that fatalism was mainly mediated through attitudes, whereas comparative optimism had both direct and mediated effects. The findings are discussed in relation to the role of these social psychological factors for COVID-19 mitigation.

## Introduction

During the rapid worldwide spread of the severe acute respiratory syndrome coronavirus 2 (SARS-CoV-2) in the early phases of the year 2020, several sanitary protocols were introduced. These protocols were established to contain the spread of the virus in the lack of established first-line treatment and an effective vaccine. Two of the most important protocols were proper hand hygiene in general, and in public places specifically, and keeping a physical distance from other individuals. The World Health Organization (2020) has pointed to individual adherence to these protocols as critical to control the reproduction rate of the virus. Research has suggested that the virus tends to spread faster in densely populated areas (Hamidi et al., 2020; Wheaton and Thompson, 2020), and urban density may also negatively influence the possibility of proper social distancing. Tehran in Iran was one of the most severely affected areas in the early stages of the pandemic, and the figures have remained consistently high, with approximately 8,000 incidents per day as of mid-March 2021 (Johns Hopkins University Centre, 2021). This renders Tehran an interesting case for coronavirus disease 2019 (COVID-19) research.

Underlying evaluations and beliefs are considered instrumental in shaping individual behavior (Ajzen, 1991; Stern and Dietz, 1994). Fatalism refers to a psychological tendency to perceive events as uncontrollable, predetermined, and outside individual control (Ngueutsa and Kouabenan, 2017; MclLroy et al., 2020). People with fatalistic beliefs tend to explain events by factors such as fate or bad luck, which are unamendable by human action and induces passivity when confronted with health risks (Simsekoglu et al., 2013). Some studies have found a correlation between fatalistic beliefs and religion (Ruiu, 2013), and fatalism may be particularly relevant to behavior in countries where religion places strong guidelines on individual behavior. Research has shown that fatalism may be associated with less precaution when it comes to protective health behaviors ranging from precautious driver behavior (Kouabenan, 1998; Peltzer and Renner, 2003; Nordfjaern et al., 2012) to more general health habits such as healthy eating (Welch and Ellis, 2018) and cervical screening (Marlow et al., 2018). However, some studies did not reveal any significant associations between fatalism and health behavior (e.g., Jones et al., 2016; Moss et al., 2019).

The COVID-19 protocols are, to a large extent, based on the assumption that the infection curves may be affected by individual action. As such, control perceptions inherent in fatalism may be of importance for adherence to the COVID-19 protocols. One unpublished study indirectly examined fatalism in relation to COVID-19 sanitary protocols. Akesson et al. (2020) carried out an online experiment with three arms and concluded that those who perceived the SARS-CoV-2 to be more infectious were less likely to conduct social distancing. Important limitations of this study were that an explicit measure of fatalism was not incorporated, and the study solely focused on one of the sanitary protocols.

Another social–psychological belief with potential importance for adherence to the COVID-19 sanitary protocols is comparative optimism (i.e., unrealistic optimism or optimism bias). Comparative optimism refers to a bias where people overestimate their chances of desirable outcomes and underestimate their probability of negative outcomes relative to their peers (see Shepperd et al., 2002; Chambers and Windschitl, 2004; for extensive reviews). The evidence regarding the role of unrealistic optimism for health-promoting behavior is rather mixed. Some studies have suggested that comparative optimism may facilitate risk behavior (e.g., Radcliffe and Klein, 2002; Jefferson, 2006; Fallah Zavareh et al., 2018) and attributed this to perceptions about less vulnerability. Meanwhile, Taylor and Brown (1988) concluded decades ago that unrealistic optimism could be a characteristic of well-functioning mental health and may be particularly adaptive under threatening or uncertain circumstances. The bias may be correlated with self-esteem (Taylor et al., 2003) and could induce self-fulfilling prophecies, as people may try harder to achieve desirable outcomes and goals. Aligned with this assumption, Menon et al. (2009) reported that in scenarios where certain actions give individuals a high perceived control over the outcomes, people tend to become more optimistic on their own behalf and conduct the actions needed to achieve desirable outcomes.

### Aims and Hypotheses of the Study

**FIGURE 1:**
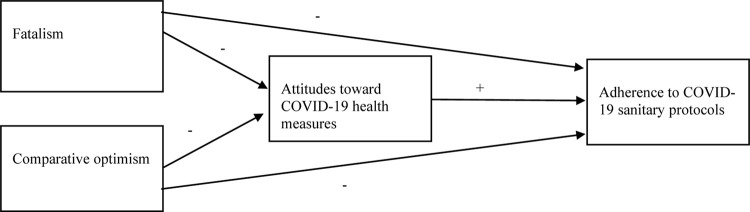
Heuristic working model of the study. −Hypothesized negative association +Hypothesized positive association.

The study aimed to investigate the relative roles of fatalism and comparative optimism for attitudes toward COVID-19 health measures and adherence to COVID-19 sanitary protocols. As shown in Figure 1, aligned with social cognitive theory (e.g., Ajzen, 1991), we tested whether fatalism and comparative optimism directly predicted adherence to COVID-19 sanitary protocols or whether these were cognitively mediated through evaluations of COVID-19 health measures (i.e., attitudes toward COVID-19 health measures). Based on the research reviewed earlier, we hypothesized that fatalism and comparative optimism would be associated with less health-promoting attitudes toward COVID-19 health measures and lower adherence to the protocols. Aligned with social cognition theory, we assumed that attitudes and adherence would be positively associated.

## Materials and Methods

### Procedure

The results are based on a web survey carried out at Kharazmi University in Tehran in 2020. The survey was conducted from April to June 2020, a period where the country was in full lockdown. A questionnaire was devised by an international research group consisting of experts in psychology and epidemiology, and the questionnaire was uploaded to Google Docs. The survey included an information letter about data confidentiality and integrity. The link to the survey was distributed at online university groups and forums. The university administration and academic staff were mobilized and encouraged to share the link after online lectures. A snowballing approach was also used, such that the respondents were requested to share the link with their acquaintances and social network with the inclusion criterion that these were also students at Kharazmi University. As there are no formal ethical institutional review boards established in Iran and because the data analysis was conducted in Norway, the project was reviewed by the Norwegian Centre for Research Data (reference 291734). The Norwegian Centre for Research Data approved that processing and data analyses could proceed because the data delivered from Iran were anonymized.

### Sample Characteristics

The final sample consisted of 370 individuals. Among these, 57% (*n* = 211) were females and 43% (*n* = 159) males. The mean age of the respondents was 22.12 years (SD = 3.18, range = 19–39 years). A total of 30.08% (*n* = 114) reported their household income to be lower or much lower than the average in the city where they studied, whereas 49.50% (*n* = 143) reported the income to be the same, and 19.70% (*n* = 73) reported it to be higher or much higher.

### Questionnaire and Measurement Instruments

The questionnaire was part of a larger survey investigating transportation mode used during the onset of the COVID-19 pandemic in Iran. The questionnaire included several validated scales originally in English, which were translated to Farsi language by researchers with high proficiency in both languages. Demographic characteristics used to describe the current sample included each respondent’s sex and age. We also asked the respondents to report their household income compared with the average income in the city where they studied. This item was scored on a Likert scale ranging from (1) much lower to (5) much higher. The two lowest and two highest anchors were merged to facilitate interpretation.

Fatalism was measured by an adopted version of the multidimensional fatalism instrument reported in Esparza et al. (2015) and MclLroy et al. (2020). The instrument used by MclLroy et al. (2020) consisted of 30 items. We included 14 items loaded on three dimensions across different countries with cultural and geographic variation in their measurement model. These three dimensions included “general fatalism” (e.g., “If bad things happen, it is because they were meant to happen”), “internality” (e.g., “What happens in the future mostly depends on me”), and luck (e.g., “When I get what I want, it’s usually because I am lucky”). The five items that loaded on the “divine control” dimension (e.g., “God controls everything good and bad that happens to a person”) in MclLroy et al. (2020) were excluded from the current study due to the sensitivity of religious issues in Iran and that items addressing such issues may negatively impact the response rates in this setting. The 14 fatalism items were scored on a Likert scale ranging from (1) strongly disagree to (5) strongly agree.

Comparative optimism was measured by asking the respondents to compare themselves with others of the same age and sex in terms of cautiousness, probability, and vulnerability of being infected with the SARS-CoV-2 virus. The items were adjusted toward the COVID-19 circumstances but were based on instruments of comparative optimism used in previous work (Delhomme, 1991; Martha et al., 2010; Fallah Zavareh et al., 2018). The six items of comparative optimism were scored on a Likert scale ranging from (1) much worse to (5) much better. Based on factor analysis, Fallah Zavareh et al. (2018) demonstrated that the instrument is unidimensional.

A nine-item instrument was used to measure attitudes toward COVID-19 health measures. This instrument included items that measured respondents’ evaluations about COVID-19 health measures, such as “Coronavirus spread can only be avoided if human behavior is radically changed” and “I feel a personal responsibility for others not to catch the Coronavirus.” The items in this scale were measured on a Likert scale ranging from (1) strongly disagree to (5) strongly agree. Some items were reverse coded to make higher scores reflect health-promoting evaluations about COVID-19 health measures.

Finally, a seven-item index was used to tap information about adherence to COVID-19 sanitary protocols. This instrument asked respondents to report how frequently they conducted different sanitary actions when in public places. The actions covered common sanitary measures recommended by the World Health Organization (2020), such as using disinfectives, wearing a face mask, handwashing, avoiding public places in peak hours, avoiding touching surfaces, and keeping distance from other individuals. The items were scored from (1) never to (5) very often.

An overview of all measurement items included in the current study is provided in Appendix Table 1.

### Statistical Procedures

Descriptive statistics were used to describe the sample and to reveal mean scores and standard deviances on the study variables. Spearman’s correlation coefficients were used to investigate bivariate associations between the variables. Cronbach’s α coefficients and average corrected item-total correlations were calculated to reveal reliability indices for the scales and indexes. As the measures of attitudes toward COVID-19 health measures and adherence to COVID-19 sanitary protocols represented new instruments, these were the first subject to principal component analysis (PCA) with iteration, Kaiser criterion, and varimax rotation. Kaiser–Meyer–Olkin (KMO) and Bartlett’s test of sphericity were used as assumption tests for the PCA. IBM SPSS Statistics Version 23 was used for these analyses.

To test the hypothesized model (Figure 1), structural equation modeling (SEM) was conducted using the IBM SPSS AMOS version 23 software. SEM was conducted in a two-step sequence as suggested by, for instance, Anderson and Gerbing (1988) and Bamberg (2006). In the first step, a confirmatory factor analysis (measurement model) was fitted for each individual measurement instrument. Modifications of the measurement models in terms of weak factor loadings and amendments based on the modification indices were carried out for models that showed improvement potentials in the initial specification. We also tested the overall fit of the complete measurement model with all the instruments incorporated into the same confirmatory factor analysis. After the measurement model was fitted, we added structural relations between the latent factors as specified in Figure 1. For simplicity and brevity, we only display the outcome of the structural modeling in *Results*. The model also adjusted for correlations between all the latent predictors, but these are not shown to facilitate interpretation. The fit indices used for both the measurement model and SEM were the root mean square error of approximation (RMSEA) and comparative fit index (CFI). CFI values around 0.90–0.95 and RMSEA values below 0.08 indicate a tolerable fit between the model and data (Kim and Bentler, 2006; Ho, 2013). To investigate whether fatalism and comparative optimism had mediated associations to adherence, we also calculated direct, indirect, and total effects. Variance inflation factor (VIF) values were examined to detect potential multicollinearity in the model. Collinearity issues may be present when the VIF values exceed 4.00 with tolerance below 0.20 (Hair et al., 2010).

### Factor Structure and Reliability of the Instruments

Assumption tests showed that both the attitudes toward COVID-19 health measures (KMO = 0.91, χ^2^ = 2441.18, *p* < 0.001) and adherence to COVID-19 sanitary protocols (KMO = 0.90, χ^2^ = 1289.30, *p* < 0.001) were suited for PCA. The nine items in the attitudes toward COVID-19 health measures segmented into a unidimensional structure with one dimension, which explained approximately 61.42% of the total variance. The dimension loadings ranged from 0.55 to 0.91. Similarly, the seven items tapping adherence to COVID-19 sanitary protocols segmented into one dimension, which explained 61.14% of the total variance. The dimension loadings in this instrument ranged from 0.75 to 0.83.

A confirmatory factor analysis on the fatalism instrument reflected improvement potential of the three-factor solution (χ^2^ = 344.62, df = 74, RMSEA = 0.100, CFI = 0.92). One item (“People die when it is their time to die, and there is not much that can be done about it”) had a rather low factor loading of 0.52. When this item was excluded from the model estimation, the model reflected adequate fit to the data (χ^2^ = 200.59, df = 62, RMSEA = 0.078, CFI = 0.95).

The initial measurement model of the unidimensional comparative optimism reflected relatively poor fit (χ^2^ = 182.94, df = 9, RMSEA = 0.229, CFI = 0.85). One item with a factor loading of 0.33 (vulnerability compared to others) was removed and two residual correlations were added based on the modification indices. After these amendments the model reflected good fit to the data (χ^2^ = 4.70, df = 3, RMSEA = 0.039, CFI = 0.99).

The hypothesized unidimensional factor of attitudes toward COVID-19 health measures had improvement potential (χ^2^ = 236.89, df = 27, RMSEA = 0.145, CFI = 0.91). Based on modification indices, one residual correlation was added to the model, and the model was reestimated. The re-specified measurement model had good fit to the data (χ^2^ = 65.34, df = 26, RMSEA = 0.064, CFI = 0.98).

The unidimensional instrument of adherence to COVID-19 sanitary protocols also revealed improvement potential (χ^2^ = 75.15, df = 14, RMSEA = 0.109, CFI = 0.95). Based on the modification indices, the model was re-specified with one residual correlation. After the re-specification, the model reflected good fit (χ^2^ = 30.10, df = 13, RMSEA = 0.060, CFI = 0.99).

Finally, we tested a complete measurement model with all factors described earlier in one coherent model. The model had good fit to the data (χ^2^ = 1198.26, df = 520, RMSEA = 0.059, CFI = 0.92). As shown in Table 1, all the fitted factors had satisfactory reliability indices.

**TABLE 1:** Reliability and internal consistency of the instruments.

**Factors**	**Mean (SD)**	**Scale**	**Number of items**	**Cronbach’s α (aic)**
*Fatalism*		1–5		
General fatalism	2.20 (0.85)		5	0.877 (0.71)
Internality	3.97 (0.95)		3	0.915 (0.83)
Luck	2.49 (0.84)		5	0.885 (0.73)
*Comparative optimism*	3.46 (0.69)	1–5	5	0.863 (0.69)
*Attitude toward COVID-19 health measures*	3.87 (0.79)	1–5	9	0.916 (0.71)
*Adherence to COVID-19 sanitary protocols*	4.05 (0.81)	1–5	7	0.891 (0.69)

*aic, average corrected item-total correlation.*

## Results

### Bivariate Associations Between the Study Variables

Correlations between the factors of fatalism, comparative optimism, attitudes toward COVID-19 health measures, and adherence to COVID-19 sanitary protocols are shown in Table 2. As expected, general fatalism was associated with less internality and higher scores on the luck factor of fatalism. General fatalism was also associated with less health-promoting attitudes toward COVID-19 health measures. The internality dimension of fatalism was related to higher comparative optimism, more health-promoting attitudes toward COVID-19 measures, and stronger adherence to sanitary protocols. The luck dimension was associated with less health-promoting attitudes toward COVID-19 health measures. On the other hand, comparative optimism was positively correlated with health-promoting attitudes and stronger adherence. Finally, attitudes toward COVID-19 health measures were positively associated with adherence to COVID-19 sanitary protocols.

**TABLE 2:** Correlations between the study variables.

**Indicator**	**1**	**2**	**3**	**4**	**5**	**6**
1. General fatalism	–	**−0.16**	**0.56**	0.03	**−0.19**	**−**0.06
2. Internality		–	**−**0.06	**0.23**	**0.48**	**0.29**
3. Luck			–	0.06	**−0.17**	**−**0.02
4. Comparative optimism				–	**0.40**	**0.44**
5. Attitudes toward COVID-19 health measures					–	**0.48**
6. Adherence to COVID-19 sanitary protocols						–

*Significant (*p* < 0.001) correlations in bold.*

### Predictors of Adherence to the Coronavirus Disease 2019 Sanitary Protocols

**FIGURE 2:**
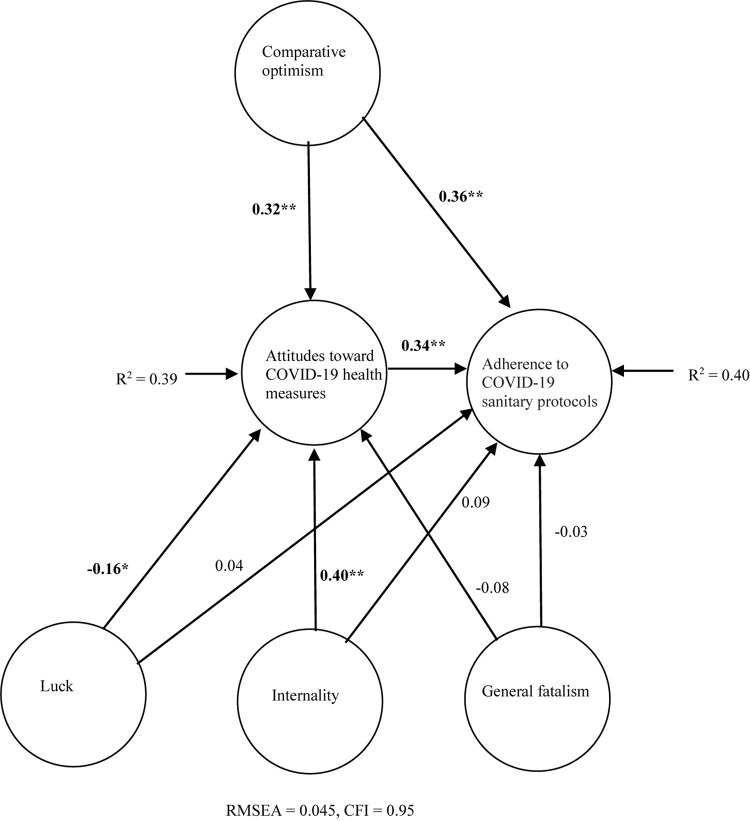
Predictors of adherence to COVID-19 sanitary protocols. Significant standardized path coefficients in bold. *p < 0.05, **p < 0.001. Model controlled for all possible correlations between the predictors (fatalism factors and comparative optimism). Manifest variables not shown to facilitate interpretation.

VIF-values ranged from 1.32 to 1.69, and the tolerance values were between 0.59 and 0.76. This suggests that multicollinearity was not a substantial issue in the specified structural model. The fit indices suggested that the hypothesized structural equation model had very good fit to the data (χ^2^ = 881.64, df = 508. RMSEA = 0.045, CFI = 0.95). As shown in Figure 2, fatalism and comparative optimism explained 39% of the variance in attitudes toward COVID-19 health measures and 40% of the variance in adherence to COVID-19 sanitary protocols. Comparative optimism was the factor with the strongest positive multivariate association with both attitudes toward COVID-19 health measures and adherence to sanitary protocols. Among the fatalism factors, internality was strongly positively associated with health-promoting attitudes, whereas the luck factor was slightly associated with less health-promoting attitudes. Attitudes toward COVID-19 health measures were positively associated with adherence to COVID-19 sanitary protocols. The overall lack of direct effects of fatalism (Table 3) suggests that internality is indirectly associated with adherence through attitudinal mediation. Comparative optimism both had an indirect and direct relation to adherence, but the direct effect was substantially stronger than the mediated effect. Overall, comparative optimism seems to be of greater importance for adherence than fatalism.

**TABLE 3:** Direct, indirect, and total effects of fatalism and comparative optimism on adherence to COVID-19 sanitary protocols.

**Standardized effect**	**General fatalism**	**Internality**	**Luck**	**Comparative optimism**
Direct effect	**−**0.03	0.09	0.04	0.36*
Indirect effect (through attitudes)	**−**0.03	0.14*	**−**0.05*	0.11*
Total effect	**−**0.06	0.23*	**−**0.01	0.47*

*Statistically significant at 95% confidence interval.

## Discussion

The current study has shown that the social psychological factors of fatalism and comparative optimism may be of importance for adherence to the COVID-19 sanitary protocols. The suggested model explained approximately 40% of the total variance in attitudes toward COVID-19 health measures and adherence to the sanitary protocols. Aligned with the hypothesis, high internality was related to stronger protocol adherence, whereas high scores on the luck factor were slightly associated with less adherence. Fatalism was mainly associated with adherence through affecting how people cognitively evaluate COVID-19 health measures (i.e., attitudes toward COVID-19 health measures). Opposing the postulated hypothesis, comparative optimism was related to stronger adherence to the COVID-19 sanitary protocols. This variable had both direct and mediated effects. As expected, health-promoting attitudes toward COVID-19 health measures significantly predicted stronger adherence to the protocols.

The internality dimension of fatalism was found to affect adherence, mainly through attitudinal mediation. This implies that perceptions of having personal control over events facilitate more health-promoting cognitions about COVID-19 health measures, which in turn may promote protocol adherence. This adds to the evidence base showing that having an internal locus of control promotes health behavior (e.g., Steptoe and Wardle, 2001; Gale et al., 2008). However, the findings did not yield support to the assumption that internality has a direct effect on behavior, as reported in the Thai and Chinese samples in MclLroy et al. (2020). Diverging sampling contexts and outcome variables may constitute issues when comparing findings across studies. MclLroy et al. (2020) focused on samples from East Asia, Western Europe, and Sub-Saharan Africa. They also focused on rather specific health outcomes, namely risk-taking behavior in road traffic.

Somewhat surprisingly, the findings did not yield support to the growing evidence base that comparative optimism is associated with less health-promoting behavior and cognitions (see, e.g., Radcliffe and Klein, 2002; Jefferson, 2006; Fallah Zavareh et al., 2018). However, the results align with the research domain derived from the early experiment by Taylor and Brown (1988), arguing that optimism bias under high personal-control conditions may indeed facilitate more health-promoting behavior (Menon et al., 2009). A possible explanation is that people who are optimistic about their own ability to contain the virus may be more willing to undertake the individual behavior required to avoid infection. This should be interpreted with caution, however, as adherence to protocols also could promote optimism (i.e., endogeneity). This assumption is in accordance with self-perception theory (Bem, 1967), where personal behavior is thought to be highly relevant for how people cognitively evaluate the target behavior. The present study design does not allow us to exclude this opportunity, although such an association is generally challenged by social cognition theory such as the theory of planned behavior (Ajzen, 1991) and the health belief model (Janz and Becker, 1984). Experimental studies need to be devised to identify further the causal nature of comparative optimism on adherence to COVID-19 protocols.

Some limitations of the current study should be noted. The study was based on self-reports that could be subject to social desirability bias. Although the tested structural relations were based on psychological theory, the design was cross-sectional, which precludes the possibility of decisive causal inferences. The sample was based on university students and cannot be generalized to the general Iranian public. University students may report less fatalism than the general public, and they may also report higher optimism because they are young, which by itself constitutes a protective factor in terms of COVID-19 prognosis (Dowd et al., 2020). Finally, it is noted that the opportunity to conduct physical distancing may be challenging in crowded cities in middle-income countries, such as Tehran. The use of protective gear such as gloves and disinfectives is also subject to costs and affordability. Cultural differences in the perceived relative importance of social structures, such as family members and the authorities, may further influence adherence to COVID-19 health protocols (see, e.g., Biddlestone et al., 2020). Future studies could aim to incorporate socioeconomic status, cultural variables, and urban form variables in the analytical framework.

## Conclusion

Because COVID-19 vaccination currently is in early phases in most countries, mitigation of the pandemic depends on individual preventive behavior, such as adherence to sanitary protocols. The present study represents one of the first attempts to test the relative role of social psychological factors for adherence in a high-risk urban context. The findings suggest that the control perceptions inherent in fatalism and comparative optimism seem important for adherence. Internality/internal control locus seems to promote adherence to the COVID-19 protocols, at least indirectly through attitudinal mediation. Comparative optimism was associated with stronger adherence to the protocols both directly and through attitudinal mediation. Further studies should aim to identify the causal nature of these variables with adherence through experimental research designs.

## Data Availability Statement

The raw data supporting the conclusions of this article will be made available by the authors, without undue reservation.

## Ethics Statement

The studies involving human participants were reviewed and approved by Norwegian Centre for Research Data. Written informed consent for participation was not required for this study in accordance with the national legislation and the institutional requirements.

## Author Contributions

TN, MM and MFZ contributed to the methodology and questionnaire design. MFZ conducted the data collection. TN analyzed the data and wrote the first draft of the manuscript. MM and MFZ provided intellectual input to the manuscript. All authors have read and approved the final version submitted for publication.

## Conflict of Interest

The authors declare that the research was conducted in the absence of any commercial or financial relationships that could be construed as a potential conflict of interest.

## Appendix

**TABLE 1A:** Descriptives of items included in the study.

**Factors**	**Mean (SD)**	**Number of items**
*Fatalism*		14
If bad things happen, it is because they were meant to happen	2.22 (1.01)	
Life is very unpredictable, and there is nothing one can do to change the future	2.18 (1.03)	
If something bad is going to happen to me, it will happen to me no matter what I do	2.04 (0.97)	
There is no sense in planning a lot; if something good is going to happen, it will	1.98 (1.02)	
People die when it is their time to die, and there is not much that can be done about it	2.87 (1.27)	
I have learned that what is going to happen will happen	2.58 (1.16)	
What happens to me in the future mostly depends on me	4.01 (1.05)	
My own actions determine my life	3.98 (1.06)	
I feel that when good things happen, they happen as a result of my own efforts	3.91 (0.97)	
When good things happen to people, it is because of good luck	2.60 (0.96)	
When I get what I want, it is usually because I am lucky	2.51 (0.95)	
The really good things that happen to me are mostly because of luck	2.25 (0.95)	
Some people are simply born lucky	2.59 (1.12)	
How successful people are in their jobs is related to how lucky they are	2.49 (1.09)	
*Comparative optimism*		6
Competence	3.39 (0.77)	
Cautiousness	3.55 (0.88)	
Quality of preventive measures	3.52 (0.88)	
Probability of infection	3.36 (0.93)	
Vulnerability	3.24 (0.98)	
General abilities	3.47 (0.83)	
*Attitude toward COVID-19 health measures*		9
Coronavirus spread can only be avoided if human behavior is radically changed	3.67 (1.12)	
To avoid the spread of coronavirus is something that is very important to me	4.07 (1.02)	
I think it is important to encourage other people to behave healthily	4.03 (1.00)	
I feel a personal responsibility for others not to catch the coronavirus	4.05 (1.01)	
It is worth an extra effort to take care of my own health against the coronavirus	3.97 (1.05)	
I feel a personal responsibility in preventing the spread of coronavirus in transport	3.96 (1.00)	
I think it is important to take care of health at all times	3.98 (1.05)	
I have good knowledge about how to avoid being infected by the coronavirus in public transport	3.49 (1.01)	
I know very well how to avoid being infected by the coronavirus	3.58 (0.98)	
*Adherence to COVID-19 sanitary protocols*		7
Keep a physical distance from other individuals	4.11 (0.90)	
Handwashing	4.42 (0.91)	
Use sanitary gloves	3.75 (1.23)	
Avoid touching surfaces	4.04 (0.97)	
Avoid using public places in peak hours	3.95 (1.06)	
Use a facemask	4.01 (1.10)	
Use disinfectives	4.08 (1.12)	
